# (R)-albuterol decreases immune responses: role of activated T cells

**DOI:** 10.1186/1465-9921-9-3

**Published:** 2008-01-14

**Authors:** Marcela A Ferrada, Erin L Gordon, Kai Yu Jen, Hong Zhen He, Xin Lu, Leesa M Barone, Sepideh Amirifeli, David L Perkins, Patricia W Finn

**Affiliations:** 1 Pulmonary and Critical Care Division, University of California San Diego, La Jolla, USA; 2 Department of Medicine, University of California San Diego, La Jolla, USA; 3 Department of Surgery, University of California San Diego, La Jolla, USA; 4 Department of Family and Preventive Medicine, University of California San Diego, La Jolla, USA; 5 Sepracor Inc., Marlborough, USA

## Abstract

Racemic albuterol is an equimolar mixture of two isomers, (R) and (S). Whether (R) and (S) isomers and the combination of both exert different effects in immune activation is not well defined. We analyzed the effects of (R+S)-albuterol, (R)-albuterol and (S)-albuterol in a murine model of allergic pulmonary inflammation and in activated T cells. Mice (C57BL/6) sensitized and aerosol challenged with the allergen ovalbumin (OVA) or phosphate buffered saline (PBS) were treated with (R)-albuterol, (S)-albuterol or (R+S)-albuterol. Following administration of (R)-albuterol, allergen induced bronchoalveolar lavage eosinophils and IgE showed a decrease, albeit not significantly by ANOVA. As T cells are important in allergic inflammation, we asked whether (R+S), (R) or (S)-albuterol might differ in effects on T cells and on the activity of the inflammatory transcription factor NF-κB. In activated T cells, (R)-albuterol administration decreased levels of inflammatory cytokines and NF-κB activity. These studies suggest that (R)-albuterol decreases cytokine secretion and NF-κB activity in T cells.

## Introduction

Allergic inflammation is characterized by enhanced T cell activation leading to the production of inflammatory cytokines and initiation of pathways such as tyrosine kinase Syk involving mast cells, eosinophils, and immunoglobulin E [1-4]. In asthma, this process leads to a phenotype characterized by bronchial inflammation and airway hyperresponsiveness. Activated T cells secrete cytokines that are pivotal in the pathogenesis of atopic asthma [5-7]. Further studies have elucidated the key role played by T cell costimulatory pathways [8,9]

The cornerstone of asthma therapy is inhaled β_2_-adrenergic agonists in combination with inhaled and systemic steroids. Conventionally, inhaled beta agonists such as albuterol induce rapid bronchodilation, yet they also demonstrate anti-inflammatory properties [10,11]. T cells possess surface β-adrenergic receptors [12] which upon stimulation activate protein kinase A (PKA) and induce cAMP, altering cytokine production. Whether beta agonists can impact allergic inflammation by regulating T cell activation remains undefined.

Beta agonists are commonly available as racemic mixtures composed of equimolar mixtures of (R)- and (S)- enantiomers. Interestingly, the pharmacokinetic properties and, at times, the biological effects of these isomers differ. (R)-albuterol binds to the β_2_-adrenergic receptor with high affinity, whereas (S)-albuterol exhibits weak binding to the β_2_-adrenergic receptor [13]. Studies of the pharmacokinetics of racemic albuterol have shown that elimination of (R)-albuterol is much more rapid than that of (S)-albuterol [14,15]. Whereas the (R)-isomer induces bronchodilation [16], (S)-albuterol may induce airway hyperresponsiveness [17]. Also, (R)-albuterol demonstrates anti-inflammatory effects in both airway smooth muscle cells and T lymphocytes, while (S)-albuterol does not [18,19]. Furthermore, β_2 _agonists may also augment surfactant secretion, decrease lung endothelial permeability, and decrease airway resistance [20].

In this study, we investigated whether albuterol isomers modulate effects on allergic responses *in vivo *in a murine model of allergic inflammation and, *in vitro*, in activated T cells. Additionally, we investigated whether activity of nuclear factor κ-B (NF-κB), which is an important transcription factor involved in the regulation of inflammatory processes including asthma, is regulated by albuterol isomers [21,22].

## Methods

### Mice

Six to 8-wk-old C57BL/6 female mice were purchased from Jackson Laboratory (Bar Harbor, ME, USA). The mice were maintained according to the guidelines of the committee on animals of the Harvard Medical School and the University of California, San Diego animal facility. Both institutions are accredited by the American Association for Accreditation of Laboratory Animal Care. All animal protocols received prior approval by the institutional review board.

### Ovalbumin Sensitization and Challenge

Mice were sensitized and challenged with the allergen ovalbumin (OVA) as previously described [9,21,23,24]. OVA mice were sensitized via intraperitoneal injection with 10 μg of chicken OVA (Sigma, St. Louis, MO, USA) and 1 mg of A1(OH)2 (alum; Sigma) in 0.2 ml of phosphate-buffered saline (PBS; Sigma), followed by a boosting injection on day 7 with the identical reagents. PBS mice received 1 mg of alum in 0.2 ml of PBS without OVA. On days 14–20, mice received aerosolized challenge with 6% OVA or PBS, respectively, for 20 min/day via an ultrasonic nebulizer (Model 5000; DeVilbiss, Somerset, PA, USA). All groups were sacrificed at day 21 and analyzed for the allergic parameters described below.

### Bronchoalveolar Lavage Analysis

Each mouse underwent bronchoalveolar lavage [25], as previously described [9,21]. Cells were resuspended in RPMI (Sigma) (5 × 10^5 ^cells/ml). Slides for differential cells counts were prepared with cytospin (Shandon, Pittsburgh, PA, USA) and fixed and stained with Diff-Quik (Dade Behring, Newark, DE, USA).

### Serum IgE

Blood was obtained by cardiac puncture on day 21. Total serum IgE levels were determined by ELISA as previously described [21]. Total serum IgE concentrations were calculated by using a standard curve generated with commercial IgE standard (BD PharMingen, San Diego, CA, USA).

### Cytokine Assays

The cytokines were assayed from supernatant with LINCOplex mouse cytokine assays (LINCO Research, St Charles, Missouri) that are bead-based multiplex sandwich immunoassays with a limit detection of less than 5 pg/ml.

### Histopathology

For histological analysis, tissue samples from the left lung were removed from the thoracic cavity and fixed in 4% paraformaldehyde and routinely processed into paraffin blocks. Paraffin sections, 5 μm thick, were cut and the tissues were screened with hematoxylin and eosin to verify the presence of at least three bronchioles per section.

### Subcutaneous Insertion of Delivery Pump

On the first day of allergen challenge (day 14) a miniosmotic pump (ALZET Model 1007D, DURECT Corporation, ALZET Cupertino, CA, USA) containing (R+S)-albuterol (R)-albuterol, (S)-albuterol or PBS was inserted subcutaneously. After the mice were anesthetized (Ketamine 100 mg/kg & Xylazine 10 mg/kg), the area of pump implantation was shaved and cleaned with alcohol. An incision of 1 cm was made between the scapulae, and pumps were inserted subcutaneously. The pumps contained (R+S)- (100 μg of each isomer in a total volume of 100 μl of PBS), (R)-albuterol, (200 μg/100 μl), (S)-albuterol (200 μg/100 μl) or PBS (1×/100 μl) and delivered at a constant rate of 1 mg/kg/day.

### Test Compounds

(R)- and (S)- albuterol were provided by Sepracor, Inc (Marlborough, MA, USA).

### Measurement of Systemic Levels of (R)- and (S)- Albuterol

To determine concentrations of (R)-albuterol and (S)-albuterol in heparinized mouse plasma, 2.0 mL of ammonium acetate buffer (pH 8.7) was added to 0.2 mL of unknown sample spiked with 20 μL of 0.03 μg/mL n-methyl albuterol internal standard solution. The samples were vortexed and put through solid phase extraction using 3-mL PBA cartridges in a vacuum manifold. Cartridges were conditioned with 2 mL of methanolic glacial acetic acid, 2 mL of methanol, 2 mL of water, and 3 mL of 0.2 M ammonium acetate buffer (pH 8.7). The sample extracts were transferred to the cartridges which contained 1.0 mL of 0.2 M ammonium acetate buffer (pH 8.7). After the samples passed through the cartridges, they were rinsed with 2 mL of 0.1 M ammonium acetate buffer (pH 8.7), 2 mL of water, 1 mL of methanol : water (50 : 50), 2 mL of methanol :water : triethylamine : ammonium hydroxide (75 : 21 : 2 : 2), 1 mL of methanol : water (50 : 50) and 1 mL of methanol. Samples were eluted with 1.5 mL of methanolic glacial acetic acid. The samples were placed under vacuum and evaporated to dryness. After adding 0.150 mL of mobile phase, each tube was vortexed briefly and transferred to injection vials. The enantiomers were resolved and quantitated on a high performance liquid chromatographic system equipped with a fluorescence detector using an Astec chirobiotic T analytical column (25 cm × 4.6 mm) and a flow rate of 1.0 mL/minute. The mobile phase consisted of acetonitrile : methanol : glacial acetic acid : diethylamine (60 : 40 : 0.3 : 0.2). Analyzed concentrations were calculated using the peak height ratio of the compound of interest to internal standard using a linear (1/concentration^2^-weighted) calibration model.

### Cells

EL4, a T cell cell line (American Type Culture Collection, Bethesda, MD) was established from a lymphoma induced in a C57BL/6 mouse by 9,10-dimethyl-1,2-benzanthracene. Murine splenocytes were isolated from C57BL/6 naïve mice and cultured in RPMI 1640 medium supplemented with 10% heat-inactivated FCS, 2 mM L-glutamine, 50 U of penicillin/ml, 50 μg of streptomycin/ml, and 50 μM 2-ME (complete medium). For activated samples, cells were cultured with Con A (5 μg/ml) and PMA (100 ng/ml) for 12 hours and then treated with (R)-albuterol (10^-6 ^M), (S)-albuterol (10^-6 ^M), or racemic albuterol [(R)-albuterol (10^-6 ^M)+(S)-albuterol (10^-6 ^M)] for the next 36 hours. For resting samples, cells were treated with Con A (5 μg/ml) and PMA (100 ng/ml), (R)-albuterol (10^-6 ^M), (S)-albuterol (10^-6 ^M), or racemic albuterol [(R)-albuterol(10^-6 ^M) + (S)-albuterol (10^-6 ^M)] for 24 hours.

### Quantitative Real-time PCR

Total RNA was isolated from EL4 cells and splenocytes with TRI Reagent (Sigma-Aldrich, St. Louis, MO). Isolated RNA was reverse transcribed with SuperScript II RNAse reverse transcriptase (Life Technologies, Carlsbad, CA). Specific primer pairs for GAPDH (housekeeping gene), IL-2, IL-6, IL-13, and IFN-γ were designed with the Primer Express software (Applied Biosystems, Foster City, CA, USA). The sequences of the forward (FW) and reverse (RE) primer pairs used in the experiments were as follows: GAPDH: TTGTGGAAGGGCTCATGACC (FW), TCTTCTGGGTGGCAGTGATG (RE) (NM008084), IL-2: GTCAACAGCGCACCCACTT (FW), TGCTTCCGCTGTAGAGCTTG (RE) (NM008366), IL-6: TTCCATCCAGTTGCCTTCTTG (FW), GAAGGCCGTGGTTGTCACC (RE) (NM008355), IL-13: AATCTGTCTGCAGGTGGGCT (FW), GGCTTCTCACTTTCATTGGCAC (RE) (NM031168), IFN-γ: AGGTGTCACAACTGCTGCCA (FW), ACACCCGAATGAGCTGCTCT (RE) (NM008337). Direct detection of the PCR product was monitored by measuring the increase in fluorescence caused by the binding of SYBR Green to dsDNA. Using 5 μl of cDNA, 5 μl of primer, and 10 μl of SYBR Green Master Mix (Applied Biosystems) per well, the gene-specific PCR products were measured continuously by means of GeneAmp 5700 sequence detection system (Applied Biosystems) during 40 cycles. Non-template controls and dissociation curves were used to detect primer-dimer conformation and non-specific amplification. The threshold cycle (C_T_) of each target product was determined and set in relation to the amplification plot of GAPDH. The C_T _is the number of PCR cycles required for the fluorescence signal to exceed the detection threshold value. The detection threshold was set to the log linear range of the amplification curve and kept constant (0.3) for all data analysis. The difference in C_T _values of two genes was used to calculate the fold difference.

### NF-κB Reporter Assay

The NF-κB promoter luciferase (pGL2) [26] and β-galactosidase reporter gene (pGK) [27] have been described previously. Each construct (1 μg) was added to EL4 cells (to 2 × 10^6^) resuspended in nucleofector solution (Amaxa Biosystems) and electroporated using the C-9 program of the nucleofector. After 24 hours cells were treated Con A (5 μg/ml) and PMA (100 ng/ml), [(R)-albuterol(10^-6 ^M) + (S)-albuterol (10^-6 ^M)], (R)-albuterol (10^-6 ^M) or (S)-albuterol (10^-6 ^M), for 24 hours. For activated samples, cells were cultured with Con A (5 μg/ml) and PMA (100 ng/ml) for 12 hours and then treated with [(R)-albuterol (10^-6 ^M)+(S)-albuterol (10^-6 ^M)], (R)-albuterol (10^-6 ^M) or (S)-albuterol (10^-6 ^M), for the next 12 hours. Cells were lysed in reporter lysis buffer (Promega). Then, 10 μl of the cell lysate was mixed with 100 μl of luciferase assay reagent (Promega), and luciferase activity was measured by a luminometer (Turner Bio Systems). Luciferase activity was normalized for transfection efficiency by β-galactosidase activity measured with Galacto-light systems according to the manufacturer's instructions (Applied Biosystems, MA). Fold activation was calculated as the ratio of luciferase versus β-galactosidase activity in experimental samples compared to media alone.

### Statistical Analysis

Analysis of variance (ANOVA) was performed by Sigma Stat software. Bonferroni correction for statistical adjustment of the p value for multiple comparisons was applied as a post-hoc analysis. Data are reported as means ± SEM. Statistical significance was defined by p < 0.05.

## Results

### Allergen-induced pulmonary inflammation is not influenced by the insertion of a miniosmotic pump

**Figure 1:**
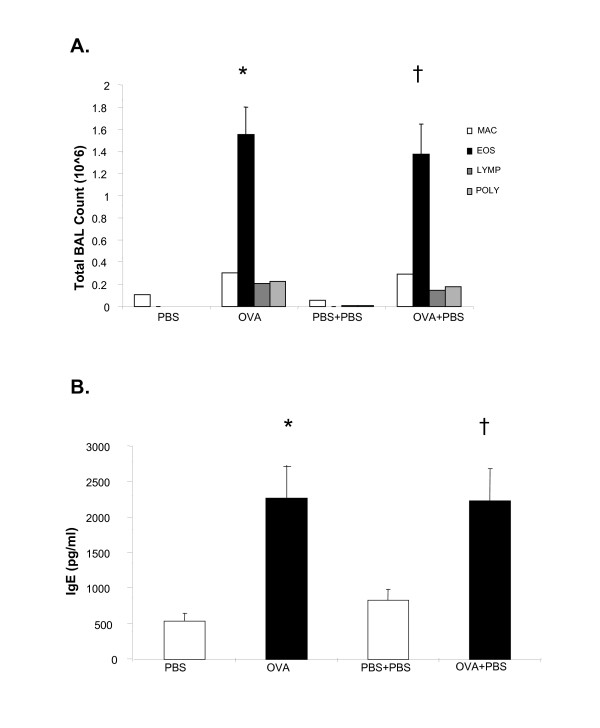
Pump insertion does not alter allergic immune responses. Mice were sensitized and challenged with ovalbumin (OVA) as described in Methods. On the first day of OVA challenge (day 14, see Methods) a miniosmotic pump containing PBS (1×/100 ul) was inserted subcutaneously and delivered a constant dose of 25 ul/day. Cell counts were determined by differential staining of cells isolated from bronchoalveolar lavage (BAL) fluid. Total serum IgE and BAL cytokines (R&D Systems) were measured by ELISA. (A) BAL eosinoplhlia; (B) total serum IgE; (C) BAL IL-13. Data is shown as mean ± SEM (n = 8–10 per group). *PBS vs OVA p < 0.01, † PBS+PBS vs OVA+PBS p < 0.01.

To analyze the effects of albuterol isomers *in vivo *in a murine model of allergic inflammation, we analyzed the potential immune effects of a delivery device for albuterol administration i.e. a miniosmotic pump inserted subcutaneously. We defined the effects of pump insertion alone on allergen-induced inflammation in a murine model. We measured allergen-induced BAL eosinophilia and total serum IgE in C57BL/6 mice following OVA sensitization and challenge and insertion of a subcutaneous miniosmotic pump containing PBS (Fig. 1). Consistent with our previous studies, OVA sensitized and challenged mice (OVA mice) demonstrated a significant increase in BAL eosinophilia and total serum IgE as compared with PBS mice (*p < 0.05, Fig 1A, B). Following the insertion of a pump containing PBS, OVA mice demonstrated a similar increase in BAL eosinophilia (†p < 0.01, Fig 1A) and total serum IgE (†p < 0.01, Fig 1B) compared to PBS mice. OVA mice compared to OVA mice that had a pump inserted (OVA+PBS) did not exhibit significant difference in BAL eosinophilia and total serum IgE.

### Administration of albuterol isomers in a pulmonary allergic model

**Figure 2:**
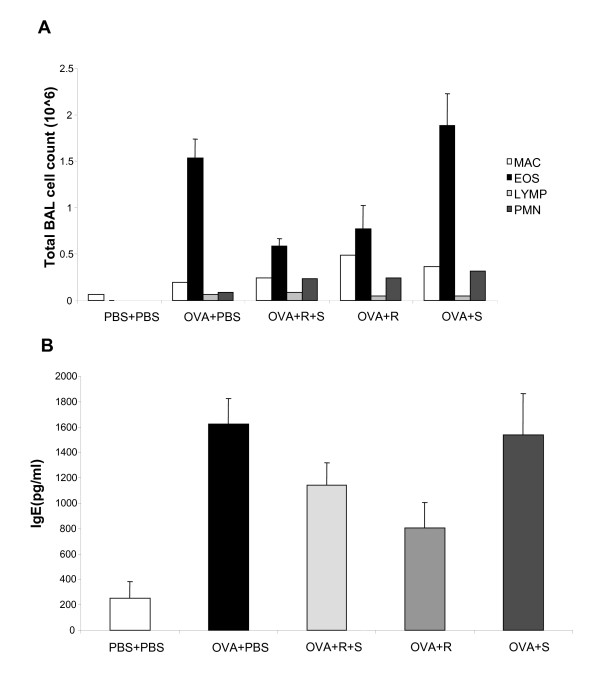
Analysis of allergic parameters following albuterol administration. (A) Mice were sensitized and challenged with OVA. On the first day of OVA challenge (day 14, see Methods) a miniosmotic pump containing (R+S), (R), (S)-albuterol or PBS was inserted subcutaneously (1 mg/kg/day). Cell counts were determined by differential staining of cells isolated from BAL fluid. Data is shown as mean ± SEM (n = 8–10 per group). (B) Total serum IgE levels were measured by ELISA. Data is shown as mean ± SEM (n = 8–10 per group).

**Figure 3:**
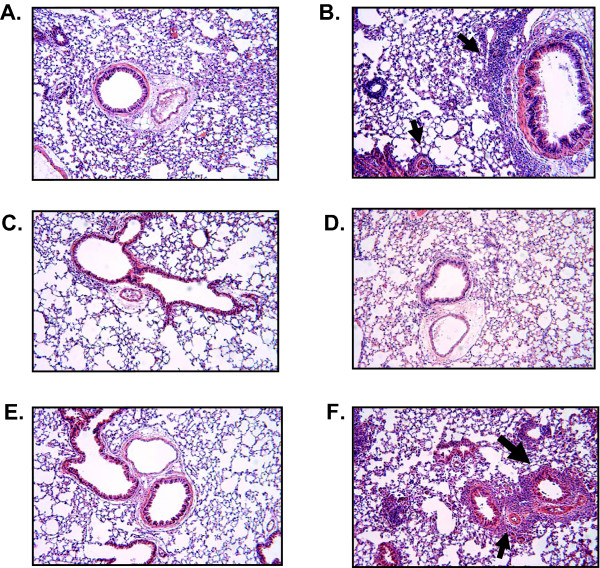
(R)-Albuterol decreases pulmonary inflammation after OVA sensitization and aerosol challenge. On the first day of OVA challenge (day 14, see Methods) a miniosmotic pump (Alzet) containing (R)- or (S)- albuterol was inserted subcutaneously (200 μg/100 μl) and delivered a constant dose of 1 mg/kg/day (25 μl/day). A) PBS+pump, B) OVA+ pump, C) PBS+(R), D) OVA+(R), E) PBS+(S) and F) OVA+ (S). These pictures are representative of two mice examined in each group. Arrows show inflammatory cells. Magnification 10×.

We next examined the effects of the albuterol isomers may influence immune responses. We measured BAL eosinophilia (Fig 2A), total serum IgE (Fig 2B) and pulmonary histology (Fig 3) in OVA mice following subcutaneous insertion of a pump containing either (R+S)-, (R)-, (S)- albuterol or PBS. As expected, OVA+PBS mice had significant increase in allergic responses compared with PBS+PBS mice (Fig 1A). OVA mice treated with (R)-albuterol demonstrated a decrease in eosinophilia (Fig. 2A) and IgE (Fig. 2B), albeit not significant by ANOVA analysis. A decrease in the pulmonary infiltrates (Fig. 3) is also observed. Serum levels of (R) and (S) albuterol were detected at day 1 and day 7 after pump insertion (not shown).

### Albuterol isomers exert a differential effect on cytokine levels following activation of splenocytes

**Figure 4:**
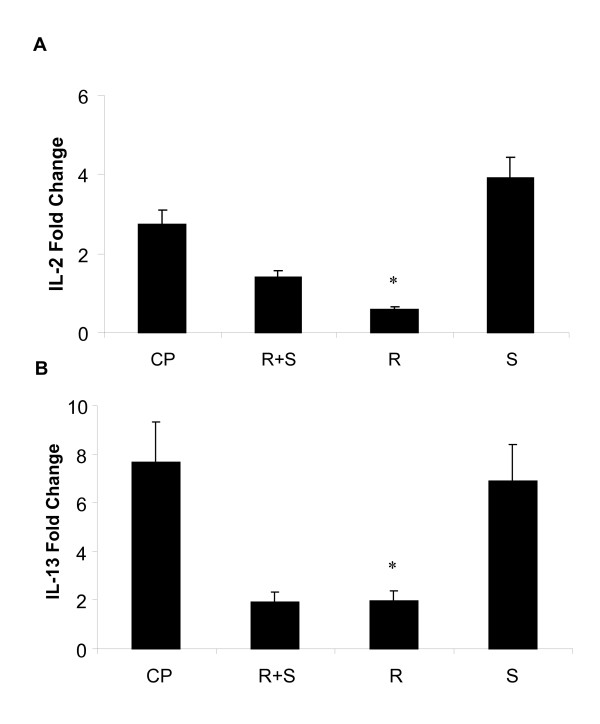
(R)-albuterol decreases cytokine levels in activated murine splenocytes. (R)-albuterol (R), (S)-albuterol (S) or racemic albuterol (R + S) at a dose (10^-6 ^M) were added to murine splenocytes pre-activated with mitogens Concanavalin A (Con A, 5 μg/ml) and phorbol myristate acetate (PMA, 100 ng/ml) (CP), (Fig 3 A, B). RNA was isolated. IL-2 and IL-13 levels were measured by real time PCR. Fold change is the ratio of stimulated to untreated sample. Data are shown as mean ± SEM (n = 3). *p < 0.05 (CP vs R).

We next determined whether albuterol effects on inflammation observed in vivo may be manifested in analysis of immune cells. Splenocytes were isolated from naïve C57BL/6 mice and activated with mitogens ConA and PMA. Activated splenocytes were incubated with (R+S), (R), (S) (10^-6 ^M) for 24 hours. IL-2 and IL-13 cytokines were analyzed by real time PCR (Fig 4A, B). (R)-albuterol significantly decreased IL-2 and IL-13 mRNA levels. There was no difference in levels of IL-6 following administration of albuterol isomers in activated cells (not shown).

### Albuterol isomers exert a differential effect on cytokine levels following activation of T Cells

**Figure 5:**
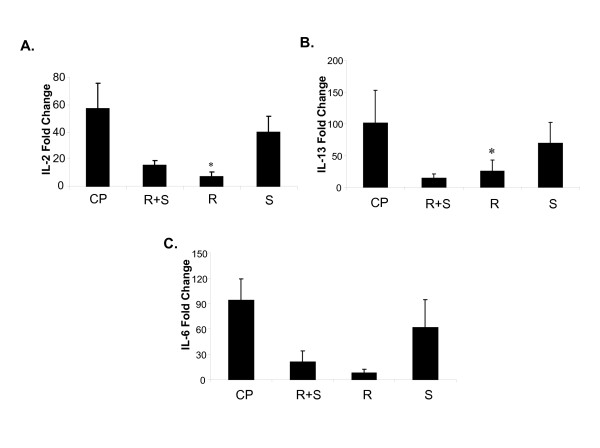
(R)-albuterol decreases cytokine mRNA levels in activated T cells. (R)-albuterol, (S)-albuterol or racemic albuterol (R + S) at a dose (10^-6 ^M) were added to T (EL-4) cells pre-activated with mitogens Concanavalin A (Con A, 5 μg/ml) and phorbol myristate acetate (PMA, 100 ng/ml) (CP). RNA was isolated. Levels of IL-2, IL-13 and IL-6 (A, B, C) were measured by real time PCR. Fold change is the ratio of stimulated to unstimulated sample. Data are shown as mean ± SEM (n = 3) *p < 0.05 (CP vs R).

**Figure 6:**
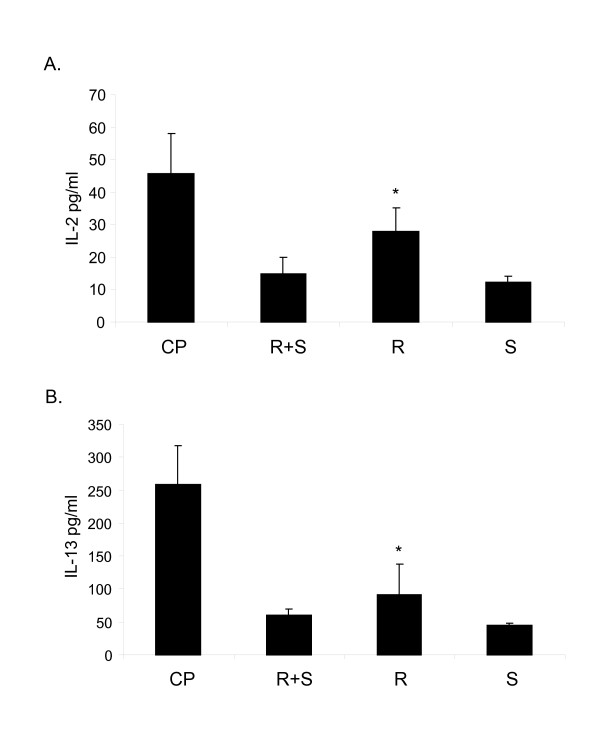
(R)-albuterol decreases cytokine protein levels in activated T cells. (R)-albuterol, (S)-albuterol or racemic albuterol (R + S) at a dose (10^-6 ^M) were added to T (EL-4) cells pre-activated with mitogens Concanavalin A (Con A, 5 μg/ml) and phorbol myristate acetate (PMA, 100 ng/ml) (CP). Supernatant was assayed for IL-2 and IL-13 cytokines by bead-based multiplex sandwich immunoassay. Data are shown as mean ± SEM (n = 3) *p < 0.05 (CP vs R).

T cells are critical for allergic inflammation [6-8,24,28]. We then investigated the effect of albuterol isomers on T cells using a T cell line (EL-4). We measured cytokine levels in both resting and activated T cells following the administration of (R+S)-,(R)- or (S)-albuterol (Figure 5A,B,C). Levels of IL-2, IL-13, IL-6 and IFN-γ were determined by real time PCR. When T cells were stimulated with mitogens ConA and PMA (CP) following by incubation with (R)-albuterol, there was a decrease in mRNA levels of IL-2, IL-13 and IL-6 (Fig. 5A, B, C). IL-2 and IL-13, but not IL-6 levels, were significantly decreased. There was no difference in levels of IFN-γ following administration of albuterol isomers in activated cells (not shown). In resting T (EL-4) cells there were no significant changes in IL-2, IL-6, IL-13 or IFN-γ levels following administration of albuterol isomers (data not shown). Thus, activated T cells demonstrate differential cytokine production when treated with albuterol isomers. We also examined cytokine secretion at the level of protein by bead-based multiplex sandwich immunoassays and found that (R)-albuterol significantly decreases IL-2 and IL-13 production (Figures 6A, 6B) (*p < 0.05).

### (R)-Albuterol decreases NF-κB activity in activated T cells

**Figure 7:**
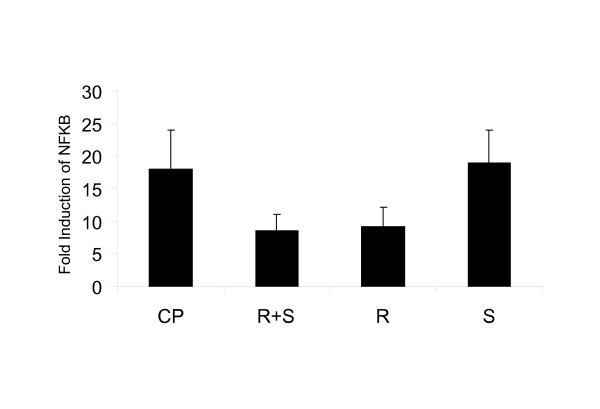
(R+S) and (R)-albuterol decrease NF-κB activity. A NF-κB luciferase reporter construct (pGL2) was cotransfected with a β-galactosidase (pGK) reporter construct by electroporation into T (EL4) cells. After transfection, (R)-albuterol (R), (S)-albuterol (S), or racemic albuterol (R+ S) were added to cells pre-activated with (ConA, 5 μg/ml) and (PMA, 100 ng/ml) (CP) and then treated with (R)-, (S)-, or (R+S)-albuterol (Fig. 6). Relative light units (RLU) were normalized to β-gal (pGK) coreporter activity. Fold induction is the ratio of RLU of stimulated to unstimulated sample. Data are shown as mean ± SEM (n = 3).

We previously demonstrated a role for the transcription factor NF-κB in allergen-induced pulmonary inflammation and the modulation of T cell subtypes [21,29]. In this study, we asked whether albuterol isomers would influence NF-κB activity in T cells. We measured NF-κB activity in resting and activated EL4 T cells by analysis of a NF-κB reporter luciferase gene construct following administration of albuterol isomers (Fig 7). Cells were activated with the T cell mitogens ConA +PMA (CP). T cells pre activated with CP, then treated with (R+S)- and (R)-albuterol display diminution of NF-κB activity when compared with cells treated with CP alone. In resting T cells, there were no changes in NF-κB activity after treatment with albuterol isomers (not shown).

## Discussion

Short acting beta agonists, including albuterol, are a mainstay of asthma therapy due to their ability to promote bronchodilation; in addition they may display anti-inflammatory properties [10,11,30,31]. Racemic albuterol contains equal concentrations of (R)- and (S)- enantiomers; yet, studies indicate that the (R)-and (S)-isomers may differ in their effects [19,32-34]. In activated T cells, we show that (R)-albuterol exhibits anti-inflammatory effects that may be mediated by alterations in NF-κB activity.

The anti-inflammatory properties of beta agonists include a reduction in proliferation of airway smooth muscle cells [18,35] as well as inhibition of cytokine-induced release of eotaxin, a potent eosinophil chemoattractant [36]. Beta agonists also inhibit the secretion of granular proteins [37] and the production of superoxide from eosinophils [32]. Prior studies suggest an anti-inflammatory effect of beta agonists on T lymphocytes. Beta agonists inhibit T cell receptor stimulated cytokine production in both human peripheral blood monocytes [38] and murine T cell clones [30] effects that may be mediated by β_2_-adrenergic receptor activation of protein kinase A (PKA) [10] and subsequent inhibition of TNF-α production and NF-κB activation [39].

(R)- and (S)- albuterol appear to differ in their pharmacological effects. While (R)- albuterol induces bronchodilation, the (S)- enantiomer shows biological effects including allergen-induced airway hyperresponsiveness in a guinea pig model and enhanced contractility in human bronchi [16]. Increases in intracellular calcium ions appear to underlie one mechanism by which the (S)-isomer may induce bronchoconstriction [20,40]. (S)-albuterol also increases the expression of the pro-inflammatory mediators, PI3 and NF-κB, in human bronchial smooth muscle cells [20], while (R)-albuterol decreases proliferation of these cells via activation of PKA and inhibition of PI-3 and NF-κB [18].

Our study examined in vivo administration of (R) + (S) albuterol isomers. To exclude the possibility that introduction of a subcutaneous delivery device (miniosmotic pump) alters immune responses, we demonstrated that insertion of a pump does not alter parameters of allergic inflammation. Previous data in a murine allergic model indicate that both (R)- and (S)-albuterol may decrease allergen-induced pulmonary inflammation and goblet cell hyperplasia [34]. Our studies and Henderson et al. exhibit different protocols, timing and methods of allergen administration. Another variable is the time of drug exposure. In Henderson's studies, the time of drug exposure appears to be 3 times longer than in our protocol [34]. Also, our murine strain is C57BL/6 while Henderson's was Balb/c. As they also did not examine (R+S) albuteroI, no comparisons can be made with regards to analysis of the (R+S) group.

T cells play an important role in the pathophysiology of asthma by modulation of inflammatory cytokines and cells [6,41,42]. Our *in vitro *studies indicate that albuterol isomers display differential effects on activated but not resting T cells. Following activation by T cell mitogens, ConA and PMA (CP), T cells treated with (R)-albuterol demonstrated decreased levels of IL-2, IL-6 and IL-13 compared to cells treated with CP alone. These findings are consistent with previous data indicating differential effects of albuterol isomers on cell proliferation and cytokine production in human peripheral blood monocytes [19].

Our findings suggest possible mechanisms for the anti-inflammatory effects displayed by albuterol isomers that may occur via T cells. Allergen-induced pulmonary inflammation is a T cell dependent process mediated by key inflammatory cytokines which promote effector pathways involving eosinophils and IgE [43]. The isomers decrease parameters of allergic inflammation in a murine model [34]. Furthermore, in activated T cells, we show that (R)-albuterol reduces the inflammatory cytokines, IL-2 and IL-13. IL-2 is a reliable marker of T cell activation [44] and IL-13 is well known for its critical role in allergen-induced inflammation [45].

Finally, our data suggest that the effects of (R)-albuterol may be mediated by alterations in the activity of the inflammatory transcription factor NF-κB. NF-κB regulates the expression of a wide range of genes involved in immune and inflammatory responses [46,47] and plays a role in the pathogenesis of asthma [21,22,48,49]. Previous studies indicate that beta agonists exert anti-inflammatory effects on monocytic cells via generation of cyclic-AMP and activation of PKA leading to a decrease in TNF-α production and NF-κB activation [39]. Also, administration of albuterol isomers induces differential expression of NF-κB in airway smooth muscle cells [18,20]. NF-κB can increase both IL-2 and IL-6 gene expression via binding to transcriptional promoter elements [25,50]. Our study indicates that (R)-albuterol decreases cytokine production and NF-κB activity in activated T cells.

## Competing interests

This work was funded by a Sepracor investigator initiated study (PWF); Leesa M. Barone is an employee from Sepracor.
